# Key dietary amino acids modulating overweight/obesity risk in Chinese children and adolescents: a machine learning analysis of a national survey

**DOI:** 10.3389/fnut.2026.1769111

**Published:** 2026-03-04

**Authors:** Qiangqiang Liu, Cheng Li, Yifan Zhang, Changqing Liu, Yiya Liu, Meina Tian, Qianrang Zhu, Yao Chen, Lianlong Yu, Hongwei Wang

**Affiliations:** 1 Department of Internal Medicine, Jinan Maternity and Child Care Hospital Affiliated to Shandong First Medical University, Jinan, China; 2 Department of Clinical Nutrition, Beijing Friendship Hospital, Capital Medical University, Beijing, China; 3 School of Public Health, Southern Medical University, Guangzhou, China; 4 Hebei Center for Disease Control and Prevention, Shijiazhuang, China; 5 Guizhou Center for Disease Control and Prevention, Guiyang, China; 6 Jiangsu Provincial Center for Disease Control and Prevention, Nanjing, China; 7 Clinical Nutrition Department, People's Hospital of Rizhao, Rizhao, China; 8 Shandong Center for Disease Control and Prevention, Jinan, China; 9 Department of Health Care, People's Hospital of Rizhao, Rizhao, China

**Keywords:** adolescents, children, dietary amino acids, machine learning, obesity, overweight

## Abstract

**Objective:**

To mitigate current research limitations, this cross-sectional study aimed to systematically evaluate the associations between dietary amino acids and overweight/obesity and to identify critical biomarkers among Chinese children and adolescents. This was achieved by integrating multiple machine learning algorithms with traditional statistical models.

**Methods:**

This study utilized data from the 2016–2019 China Children and Lactating Women Nutrition and Health Surveillance, a nationally representative survey. Participants included children and adolescents aged 6–18 years. Dietary intake was assessed using a validated food frequency questionnaire, and amino acid intakes were calculated. Four machine learning algorithms were applied to build prediction models. Model performance was evaluated via the area under the receiver operating characteristic curve (AUC). The SHapley Additive exPlanations (SHAP) method was used to interpret the optimal model and identify important features. Multivariable logistic regression models were additionally used to examine the relationship between amino acids and overweight/obesity risk.

**Results:**

A total of 8,664 participants were included. The LightGBM model showed the best predictive effect (AUC = 0.805). Both SHAP analysis and logistic regression results consistently identified leucine (OR 1.13; 95% CI 1.01 ~ 1.27), threonine (OR 1.41; 95% CI 1.22 ~ 1.63), methionine (OR 1.30; 95% CI 1.07 ~ 1.57), and cysteine (OR 0.71; 95% CI 0.59 ~ 0.84) as key amino acids associated with overweight/obesity risk. After multivariable adjustment, the intake of leucine, threonine, and methionine was positively related to the risk of overweight/obesity, whereas cysteine intake was inversely related to the risk. Restricted cubic spline analyses suggested linear relationships for these associations.

**Conclusion:**

Higher dietary intakes of leucine, threonine, and methionine are potential risk factors, while cysteine is a potential protective factor against overweight/obesity in Chinese children and adolescents.

## 1 Introduction

As the global obesity epidemic continues to intensify, childhood and adolescent obesity has become one of the most serious public health challenges worldwide (1–3). With rapid economic growth, changes in dietary structure, and decreased physical activity, the prevalence of overweight and obesity has increased rapidly in China. Obesity leads to the earlier onset of hypertension, hyperlipidemia, diabetes, and other metabolic diseases. It also negatively affects psychological development, social adaptability, and quality of life in children and adolescents (2, 4). An imbalanced dietary structure is frequently cited as a key driver of overweight and obesity. However, most existing studies focus primarily on the relationship between macronutrient intake (protein, fat, and carbohydrates) and body weight. A systematic understanding of how micronutrients, such as amino acids, contribute to overweight and obesity through complex mechanisms remains limited (5).

Amino acids play critical roles in regulating energy metabolism, hormonal signaling, adipose tissue deposition, and other physiological processes (6–9). For example, branched-chain amino acids (BCAAs) participate in body weight regulation through multiple mechanisms. These include the modulation of insulin sensitivity, alteration of fatty acid oxidation, and activation of the mTOR signaling pathway (10). Aromatic amino acids (AAAs) have also been implicated in reduced insulin sensitivity and increased adiposity (11). In addition, their microbial metabolites may disrupt systemic energy balance (12). Moreover, excessive intake of sulfur-containing amino acids (SAAs) has been suggested to promote obesity. In contrast, dietary restriction of these amino acids may improve metabolic homeostasis and support weight reduction (13, 14). Collectively, these findings indicate that specific amino acid profiles may influence body weight through pathways involving insulin sensitivity, appetite regulation, energy metabolism, and lipogenesis.

However, how different amino acids contribute to childhood overweight/obesity and the related mechanisms remain unclear. Potential synergistic or antagonistic interactions between amino acids further complicate mechanistic interpretation. Previous studies have largely focused on individual or a limited subset of amino acids, lacking a systematic evaluation of the overall amino acid feature in relation to overweight/obesity risk. In addition, the constraints of traditional epidemiological designs make it difficult to capture the complex and dynamic associations between multiple amino acids and overweight/obesity. With advances in computational science, machine learning algorithms have demonstrated superior performance in handling multidimensional data, identifying intricate feature relationships, and improving model prediction, thereby providing new methodological opportunities for nutritional epidemiology research (15, 16). On the other hand, most existing studies on amino acids and disease risk have centered on adult or elderly populations, with a paucity of large-scale, systematic investigations in children and adolescents—particularly within Chinese cohorts. Given China’s vast geography and diverse dietary patterns, regional variations may significantly modulate the relationship between amino acid intake and overweight/obesity risk. Importantly, childhood and adolescence represent critical windows for both the onset and prevention of overweight/obesity, underscoring the urgent need for nationally representative data to address these gaps.

To address the aforementioned research gaps, this research analyzed a nationally representative sample of Chinese youth aged 6–18 years and, for the first time, incorporated multiple machine learning algorithms to identify potential key amino acid biomarkers. By integrating these approaches with traditional statistical methods, we systematically evaluated the relationship between dietary amino acids and overweight/obesity risk in the study population. This work offers novel insights for understanding the effect of amino acid metabolism in the progression of pediatric overweight/obesity, and the findings may provide a scientific basis for formulating targeted dietary strategies for prevention in this population. Moreover, the study contributes methodological reference for applying artificial intelligence approaches to elucidate diet–health relationships.

## 2 Materials and methods

### 2.1 Participant selection and study design

The relevant dataset was derived from the China Children and Lactating Women Nutrition and Health Surveillance (CCLWNHS), led by the Chinese Center for Disease Control and Prevention (China CDC) during 2016–2019. Survey sites included Shandong, Jiangsu, Hebei, and Guizhou, representing eastern, southern, northern, and western regions of China. These provinces encompass diverse levels of socioeconomic development and dietary cultures, thereby capturing regional differences in dietary patterns, socioeconomic conditions, and lifestyles. Such diversity enables the study to reflect the dietary structures of children and adolescents under varying environmental contexts and provides a reliable representation of the nutritional status of children nationwide. This research was conducted in strict accordance with ethical standards, with written informed consent, and it was approved by the relevant Ethics Review Committee of China CDC (approval number: 201614).

A total of 12,976 individuals aged 6–18 years were initially enrolled in this study. Participants were excluded if they (1) were outside the age range (>18 or <6 years, *n* = 20), (2) had missing or implausible BMI values (*n* = 5), (3) lacked dietary data (*n* = 124), or (4) reported implausible energy intakes (females <500 or >3,500 kcal/day; males <700 or >4,200 kcal/day, *n* = 1765). After applying these criteria, 11,062 participants remained for analysis.

Feature selection for demographic characteristics and dietary factors covering the 20 amino acids was performed by applying the Boruta algorithm run for 100 iterations. To minimize collinearity and redundancy, only the duration of moderate-to-vigorous physical activity (MVPA), age, and sex were retained as covariates. Overweight and obese participants were then matched to normal-weight controls using propensity score matching (PSM) at a 1:2 ratio, with a caliper of 0.25. The final analytic sample comprised 8,664 individuals, including 5,776 with normal BMI and 2,888 with overweight or obesity.

### 2.2 Anthropometry and dietary assessment

Dietary data were obtained by trained investigators via a Food Frequency Questionnaire (FFQ) developed by the expert team of China CDC. The FFQ, which has undergone rigorous validation and reliability testing, was specifically designed to assess children’s dietary intake over the past month and has been widely applied in multiple large-scale national nutrition surveys in China (17–20). The questionnaire systematically covered 12 major food groups and 59 subcategories (including staple foods, soy products, vegetables, fruits, fungi and algae, meat and poultry, eggs, and dietary supplements), allowing comprehensive assessment of both intake frequency and quantity. To accurately estimate condiment consumption, a consecutive 3-day weighing method was simultaneously applied to measure household or school cafeteria use of edible oil, salt, monosodium glutamate, and other seasonings.

All investigators were equipped with standardized survey toolkits and followed a unified protocol to inquire about participants’ food consumption over the past month, including portion size, frequency, and intake for each food item that appeared in the FFQ (21). Dietary surveys were completed by the children and adolescents with the full assistance of their guardians. Nutrient and amino acid intake were assessed according to the Chinese Food Composition Table (6th Version) (22), ensuring the accuracy and completeness of dietary data.

In addition, participants’ basic demographic information, including age, sex, and region, was obtained through a standardized questionnaire. Anthropometric measurements were performed in accordance with the industry standard Methods for Anthropometric Measurements in Health Monitoring (23), and all instruments complied with national metrological certification requirements. Height and weight were measured using a metal stadiometer and an electronic scale, respectively. All measurements were conducted under a fasting state. The BMI was determined as weight (kg) divided by height squared (m^2^). Based on the Chinese national standard Screening for Overweight and Obesity (WS/T 586–2018) (24), the participants were divided into normal weight, overweight or obesity based on the relevant BMI cutoffs.

### 2.3 Covariates

We accounted for multiple covariates that may potentially impact dietary intake and overweight/obesity, including age, sex, engagement in MVPA, MVPA duration, total dietary energy, protein, carbohydrate, fat, and the ratio of energy intake from protein, carbohydrate, fat. Information on sex, age, MVPA participation, and MVPA duration was obtained through self-report.

### 2.4 Statistical analyses

Given that amino acid intake is closely related to total protein intake (11, 25, 26), we applied the nutrient density method, expressing each amino acid intake as a ratio of total protein intake to reduce the risk of multicollinearity.

In this study, the chi-square test or Fisher’s exact test was used for categorical data, while the Student’s *t*-test was applied for continuous data. Continuous data were expressed as means (standard deviations [SD]), while categorical data were expressed as frequencies (percentages [%]). To predict overweight/obesity risk, we employed four machine learning (ML) algorithms: Light gradient boosting machine (LightGBM), Extreme gradient boosting (XGBoost), Neural Networks (NN), and Naïve Bayes (NB). The predictive ability of each model was assessed via the ROC curve and AUC value. For the best-performing ML model, feature importance was evaluated via SHAP analysis to identify key influencing variables.

In building the machine learning models, we included 20 amino acids— leucine (Leu), isoleucine (Ile), valine (Val), lysine (Lys), phenylalanine (Phe), arginine (Arg), serine (Ser), threonine (Thr), methionine (Met), histidine (His), glycine (Gly), tyrosine (Tyr), cysteine (Cys), proline (Pro), aspartic acid (Asp), alanine (Ala), tryptophan (Trp), glutamic acid (Glu), aromatic amino acids (AAA), and sulfur-containing amino acids (SAA)—together with 11 covariates, including age, sex, MVPA, MVPA duration, dietary energy, protein, carbohydrate, fat, and the ratio of energy intake from protein, carbohydrate, fat.

Additionally, logistic regression was applied to determine the relation between the 20 amino acids and overweight/obesity risk. The variance inflation factor (VIF) was used to evaluate multicollinearity, and variables exhibiting a VIF ≥ 5 were excluded from the model. The final models adjusted for potential confounders, including age, sex, total energy intake, MVPA, and MVPA duration. Results were exhibited as odds ratio (OR) with 95% confidence interval (CI).

To complement the machine learning findings, the sensitivity analyses were conducted on the top 10 amino acids ranked by feature importance. These included stratified analyses, interaction tests, and nonlinear association assessments using restricted cubic splines (RCS). All the above analyses were conducted within the R statistical computing environment, utilizing relevant packages.

### 2.5 Analytical framework and predictive models

The Boruta algorithm, leveraging random forests, was used to identify features. By generating randomized “shadow features” as references, it systematically compared the original features against the shadow features, iteratively identifying those that make a significant contribution to the model. This approach is widely used for biomarker discovery and feature optimization in clinical prediction models (27, 28).

Propensity score matching is a standard technique in observational studies to tackle confounding and facilitate causal inference. Its key operation is to quantify the probability of an individual being exposed to a given factor (i.e., the propensity score), thereby balancing the distribution of covariates (e.g., age, sex) between the two groups. By mimicking the allocation mechanism of randomized controlled trials, PSM helps reduce selection bias introduced by confounding and allows for unbiased estimation of causal associations (29, 30).

LightGBM is a machine learning framework built upon gradient boosting decision trees (GBDT). It incorporates innovative techniques such as histogram-based algorithms, Exclusive Feature Bundling, Gradient-based One-Side Sampling, which substantially enhance the efficiency of conventional GBDT. LightGBM achieves a remarkable balance between computational efficiency and predictive accuracy, thereby being highly suitable for handling complex machine learning tasks (31, 32).

XGBoost is an ensemble learning technique. By iteratively training multiple trees to fit the residuals and reduce prediction errors, XGBoost is capable of capturing complex variable relationships and efficiently constructing high-performance predictive models with robustness and stability (33, 34).

Naïve Bayes is a probabilistic classification model. By analyzing the probability distribution of features under the assumption of conditional independence, NB predicts the likelihood of target classes (e.g., diseases or health outcomes). Its primary strengths lie in its simple structure, ease of implementation, and high computational efficiency, making it particularly advantageous for large-scale datasets (35, 36).

Neural Networks are a class of machine learning architectures composed of interconnected nodes that process data in layers. They contain layered arrangements of interconnected neurons, where signals are transmitted through weighted connections. Each neuron processes input via weighted summation followed by an activation function, enabling progressive feature extraction across layers and ultimately generating output predictions. NNs have demonstrated considerable value in identifying disease-related factors and predicting health risks (37, 38).

The RCS regression is designed to flexibly capture complex nonlinear associations between continuous variables and outcomes. By fitting piecewise cubic polynomials to construct smooth curves, RCS provides a more precise characterization of dose–response and other nonlinear relationships. This method combines statistical rigor with interpretability, making it particularly useful in epidemiological and clinical research (39, 40).

## 3 Results

**Figure 1:**
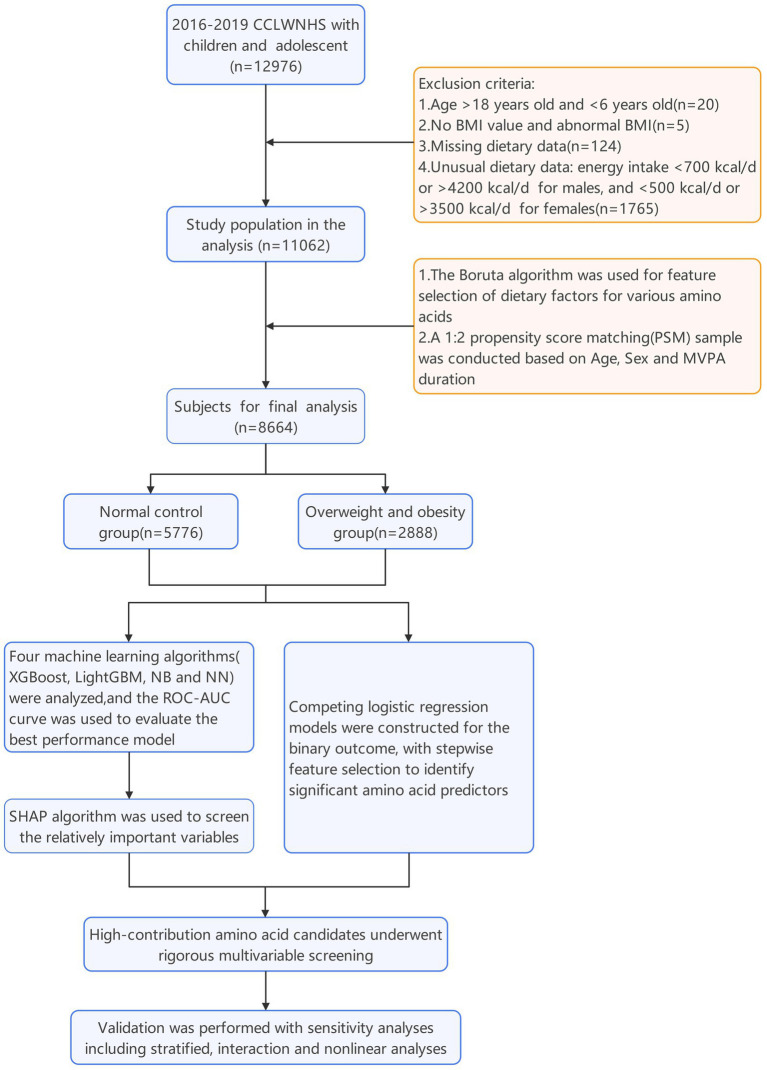
Study flow chart.

**Figure 2:**
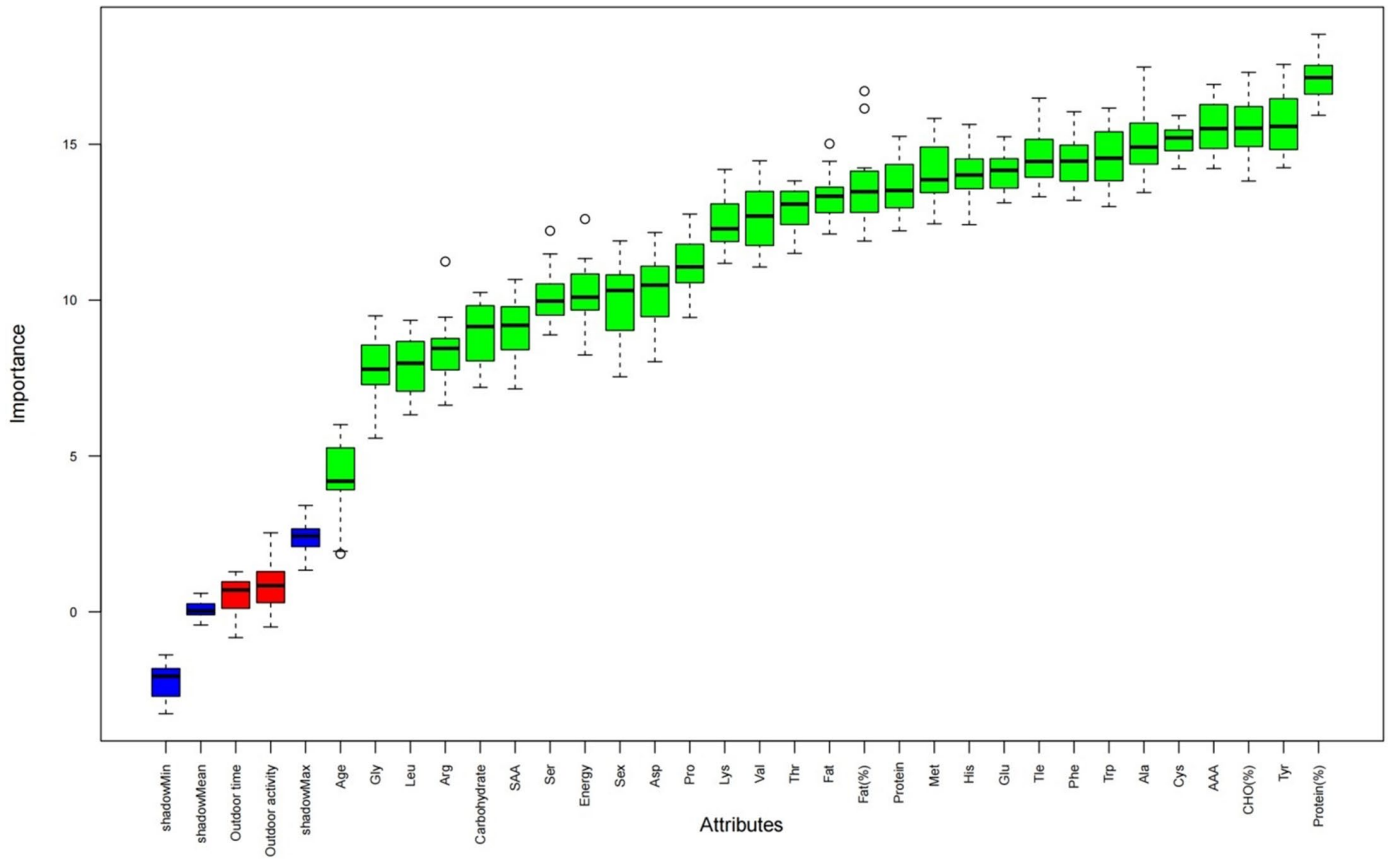
Boruta feature selection outcomes for overweight/obesity.

**Figure 3:**
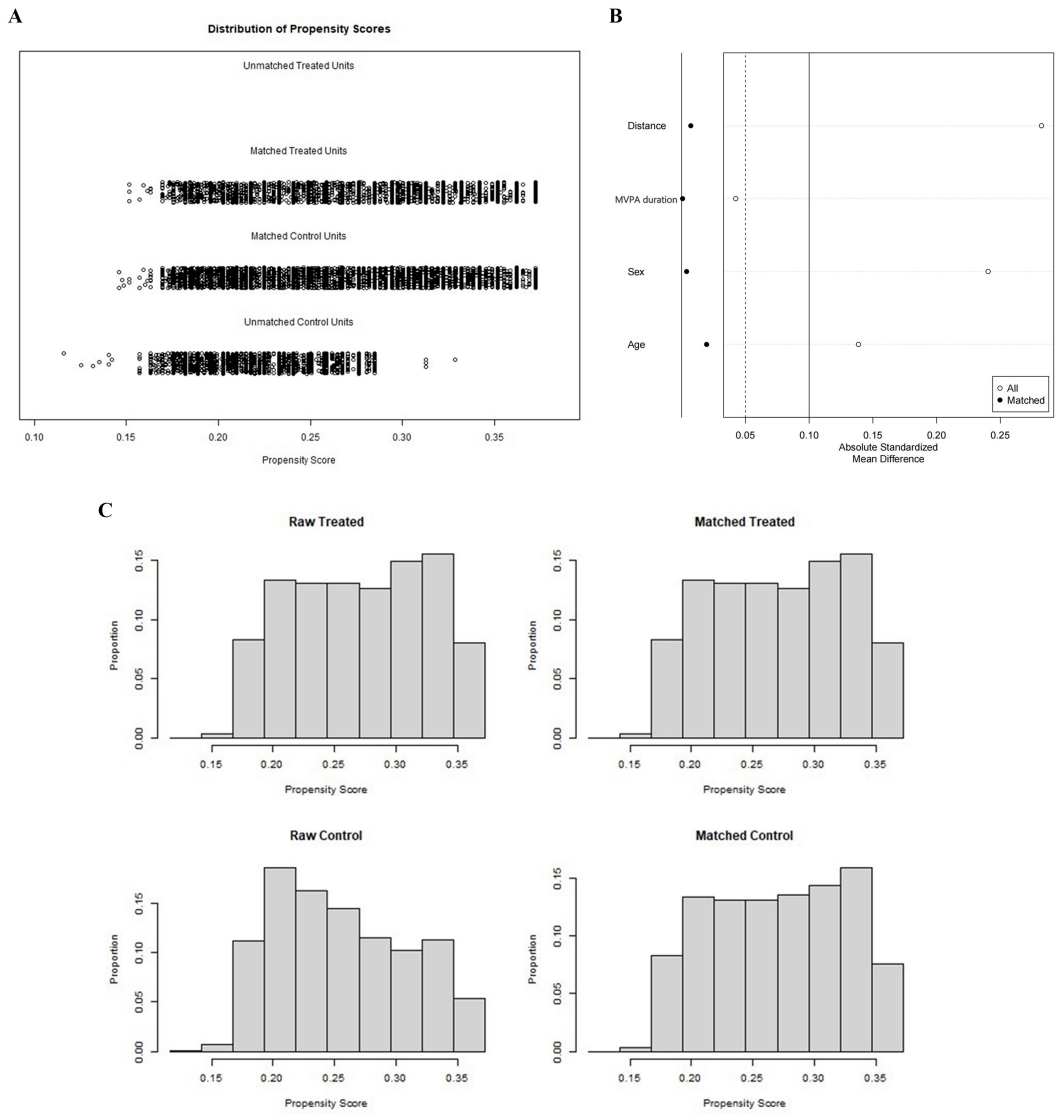
**(A)** The distribution of participants subsequent to PSM. **(B)** Love plot evaluating covariate balance before and after matching. **(C)** Histogram of sample sizes in the unmatched and matched groups.

A total of 12,976 participants were initially screened, of whom 11,062 children and adolescents qualified for inclusion after applying the eligibility criteria (Figure 1). Using the Boruta algorithm, all dietary factors were identified as relevant features associated with BMI (Figure 2). Subsequently, a 1:2 propensity score matching (caliper = 0.25) was conducted, yielding a final analytic sample of 8,664 participants, including 5,776 with normal BMI and 2,888 with overweight or obesity (Figure 3). Significant group differences were observed in protein intake, fat intake, total energy intake, and the intake percentages of Ile, Lys, Cys, Phe, Met, Thr, Tyr, Ala, and AAA, as well as the proportion of energy from the three macronutrients (all *p* < 0.05), while no significant differences were observed for other variables (Table 1).

**Table 1:** Baseline data of the participants.

Parameters	Overall (*n* = 8,664)	Normal control group(*n* = 5,776)	Overweight and obesity group (*n* = 2,888)	Statistic	*P*
Sex, *n* (%)				χ^2^ = 0.03	0.865
Female	3,523 (40.66)	2,345 (40.60)	1,178 (40.79)		
Male	5,141 (59.34)	3,431 (59.40)	1710 (59.21)		
Age(years)	10.83 ± 3.12	10.85 ± 3.12	10.79 ± 3.11	*t* = 0.86	0.392
MVPA, *n* (%)				χ^2^ = 1.15	0.283
No	1862 (21.49)	1,222 (21.16)	640 (22.16)		
Yes	6,802 (78.51)	4,554 (78.84)	2,248 (77.84)		
MVPA duration (min)	44.62 ± 42.57	44.63 ± 42.36	44.60 ± 43.01	*t* = 0.03	0.976
Ile(%)	3.77 ± 0.39	3.78 ± 0.40	3.75 ± 0.38	*t* = 3.08	0.002
Leu(%)	7.52 ± 0.39	7.52 ± 0.40	7.53 ± 0.38	*t* = −1.90	0.057
Lys(%)	4.90 ± 0.71	4.89 ± 0.70	4.94 ± 0.71	*t* = −3.38	<0.001
Ser(%)	4.47 ± 0.26	4.47 ± 0.26	4.47 ± 0.27	*t* = −0.69	0.489
Cys(%)	1.31 ± 0.26	1.32 ± 0.26	1.29 ± 0.26	*t* = 4.13	<0.001
Tyr(%)	3.36 ± 0.33	3.37 ± 0.34	3.34 ± 0.32	*t* = 4.06	<0.001
Phe(%)	4.57 ± 0.26	4.57 ± 0.26	4.56 ± 0.26	*t* = 2.88	0.004
Thr(%)	3.85 ± 0.31	3.84 ± 0.31	3.88 ± 0.31	*t* = −4.64	<0.001
Gly(%)	4.86 ± 0.52	4.86 ± 0.52	4.87 ± 0.51	*t* = −1.08	0.282
Val(%)	5.00 ± 0.32	4.99 ± 0.33	5.00 ± 0.31	*t* = −0.84	0.400
Arg(%)	5.88 ± 0.54	5.89 ± 0.55	5.87 ± 0.53	*t* = 1.88	0.061
His(%)	1.85 ± 0.28	1.85 ± 0.28	1.85 ± 0.28	*t* = −0.10	0.917
Ala(%)	5.98 ± 0.72	5.97 ± 0.72	6.01 ± 0.72	*t* = −2.40	0.017
Asp(%)	8.12 ± 0.80	8.12 ± 0.81	8.13 ± 0.79	*t* = −0.27	0.790
Glu(%)	16.86 ± 3.51	16.90 ± 3.48	16.78 ± 3.56	*t* = 1.50	0.132
Met(%)	2.10 ± 0.24	2.10 ± 0.24	2.11 ± 0.23	*t* = −2.26	0.024
Pro(%)	5.45 ± 1.11	5.45 ± 1.10	5.45 ± 1.12	*t* = 0.01	0.989
Trp(%)	1.34 ± 0.17	1.34 ± 0.17	1.33 ± 0.17	*t* = 1.52	0.128
SAA(%)	3.41 ± 0.29	3.41 ± 0.29	3.40 ± 0.30	*t* = 1.82	0.068
AAA(%)	7.93 ± 0.54	7.94 ± 0.55	7.90 ± 0.52	*t* = 3.90	<0.001
Protein(g/d)	106.13 ± 47.73	104.09 ± 47.20	110.21 ± 48.53	*t* = −5.64	<0.001
CHO(g/d)	306.16 ± 124.92	304.98 ± 125.20	308.52 ± 124.34	*t* = −1.24	0.214
Fat(g/d)	37.44 ± 25.24	36.56 ± 24.73	39.19 ± 26.13	*t* = −4.49	<0.001
Energy(kcal/d)	1986.07 ± 780.95	1965.32 ± 778.07	2027.59 ± 785.17	*t* = −3.50	<0.001
Protein(%)	22.16 ± 4.76	21.97 ± 4.79	22.56 ± 4.66	*t* = −5.53	<0.001
CHO(%)	62.15 ± 8.99	62.56 ± 9.01	61.32 ± 8.91	*t* = 6.05	<0.001
Fat(%)	16.44 ± 7.33	16.22 ± 7.25	16.88 ± 7.47	*t* = −3.96	<0.001

χ^2^: Chi-square, *t*: Student’s *t*-test.

Model 1 in Table 2 was an unadjusted model without controlling for confounders, while Model 2 was adjusted for age and sex. After testing for multicollinearity, Model 3 was constructed with full adjustment for age, sex, energy intake, MVPA, MVPA duration. Results showed that in all three logistic regression models with different levels of adjustment, Ile, Lys, Cys, Tyr, Phe, Thr, Ala, Met, and AAA were consistently significantly associated with the risk of overweight/obesity, and these associations remained statistically significant (*p* < 0.05). In Models 1 and 2, Leu and Gly showed borderline associations with overweight/obesity, but both reached statistical significance in Model 3. In Model 3, the odds ratios (ORs) for overweight/obesity associated with Ile, Leu, Lys, Cys, Tyr, Phe, Thr, Gly, Ala, Met, AAA were 0.86 (0.76 ~ 0.96), 1.13 (1.01 ~ 1.27), 1.12 (1.05 ~ 1.20), 0.71 (0.59 ~ 0.84), 0.79 (0.68 ~ 0.90), 0.80 (0.67 ~ 0.95), 1.41 (1.22 ~ 1.63), 1.10 (1.00 ~ 1.20), 1.09 (1.02 ~ 1.16), 1.30 (1.07 ~ 1.57), and 0.87 (0.79 ~ 0.94), respectively. These findings indicate a statistically significant bidirectional regulatory effect of different amino acids on the risk of overweight/obesity.

**Table 2:** Relationships of dietary amino acid intake with overweight/obesity risk in participants.

Amino acids	Model 1 OR (95% CI)	*P*	Model 2 OR (95% CI)	*P*	Model 3 OR (95% CI)	*P*
Ile(%)	0.84 (0.75 ~ 0.94)	0.002	0.84 (0.75 ~ 0.94)	0.003	0.86 (0.76 ~ 0.96)	0.008
Leu(%)	1.12 (1.00 ~ 1.25)	0.057	1.12 (1.00 ~ 1.25)	0.056	1.13 (1.01 ~ 1.27)	0.033
Lys(%)	1.12 (1.05 ~ 1.19)	<0.001	1.12 (1.05 ~ 1.19)	0.001	1.12 (1.05 ~ 1.20)	<0.001
Ser(%)	1.06 (0.90 ~ 1.26)	0.489	1.07 (0.90 ~ 1.27)	0.434	1.09 (0.92 ~ 1.29)	0.333
Cys(%)	0.69 (0.58 ~ 0.82)	<0.001	0.70 (0.58 ~ 0.83)	<0.001	0.71 (0.59 ~ 0.84)	<0.001
Tyr(%)	0.76 (0.66 ~ 0.87)	<0.001	0.76 (0.66 ~ 0.87)	<0.001	0.79 (0.68 ~ 0.90)	0.001
Phe(%)	0.77 (0.65 ~ 0.92)	0.004	0.78 (0.65 ~ 0.93)	0.005	0.80 (0.67 ~ 0.95)	0.012
Thr(%)	1.40 (1.21 ~ 1.62)	<0.001	1.40 (1.22 ~ 1.62)	<0.001	1.41 (1.22 ~ 1.63)	<0.001
Gly(%)	1.05 (0.96 ~ 1.14)	0.282	1.05 (0.96 ~ 1.14)	0.322	1.10 (1.00 ~ 1.20)	0.040
Val(%)	1.06 (0.92 ~ 1.22)	0.400	1.06 (0.92 ~ 1.22)	0.407	1.08 (0.94 ~ 1.24)	0.303
Arg(%)	0.92 (0.85 ~ 1.00)	0.064	0.92 (0.85 ~ 1.00)	0.063	0.96 (0.88 ~ 1.05)	0.342
His(%)	1.01 (0.86 ~ 1.18)	0.917	1.02 (0.87 ~ 1.19)	0.858	1.02 (0.87 ~ 1.19)	0.834
Ala(%)	1.08 (1.01 ~ 1.15)	0.017	1.08 (1.01 ~ 1.15)	0.020	1.09 (1.02 ~ 1.16)	0.009
Asp(%)	1.01 (0.95 ~ 1.07)	0.790	1.01 (0.95 ~ 1.07)	0.783	1.02 (0.96 ~ 1.08)	0.512
Glu(%)	0.99 (0.98 ~ 1.00)	0.132	0.99 (0.98 ~ 1.00)	0.155	0.99 (0.98 ~ 1.00)	0.229
Met(%)	1.24 (1.03 ~ 1.50)	0.024	1.24 (1.03 ~ 1.50)	0.026	1.30 (1.07 ~ 1.57)	0.007
Pro(%)	1.00 (0.96 ~ 1.04)	0.989	1.00 (0.96 ~ 1.04)	0.971	1.00 (0.96 ~ 1.05)	0.870
Trp(%)	0.81 (0.62 ~ 1.06)	0.128	0.81 (0.63 ~ 1.06)	0.129	0.88 (0.67 ~ 1.14)	0.328
SAA(%)	0.87 (0.74 ~ 1.01)	0.068	0.87 (0.75 ~ 1.02)	0.076	0.91 (0.78 ~ 1.06)	0.224
AAA(%)	0.85 (0.78 ~ 0.92)	<0.001	0.85 (0.78 ~ 0.93)	<0.001	0.87 (0.79 ~ 0.94)	0.001

Model 1: unadjusted. Model 2: adjusted for age, sex. Model 3: further adjusted for MVPA, MVPA duration, and energy intake.

**Figure 4:**
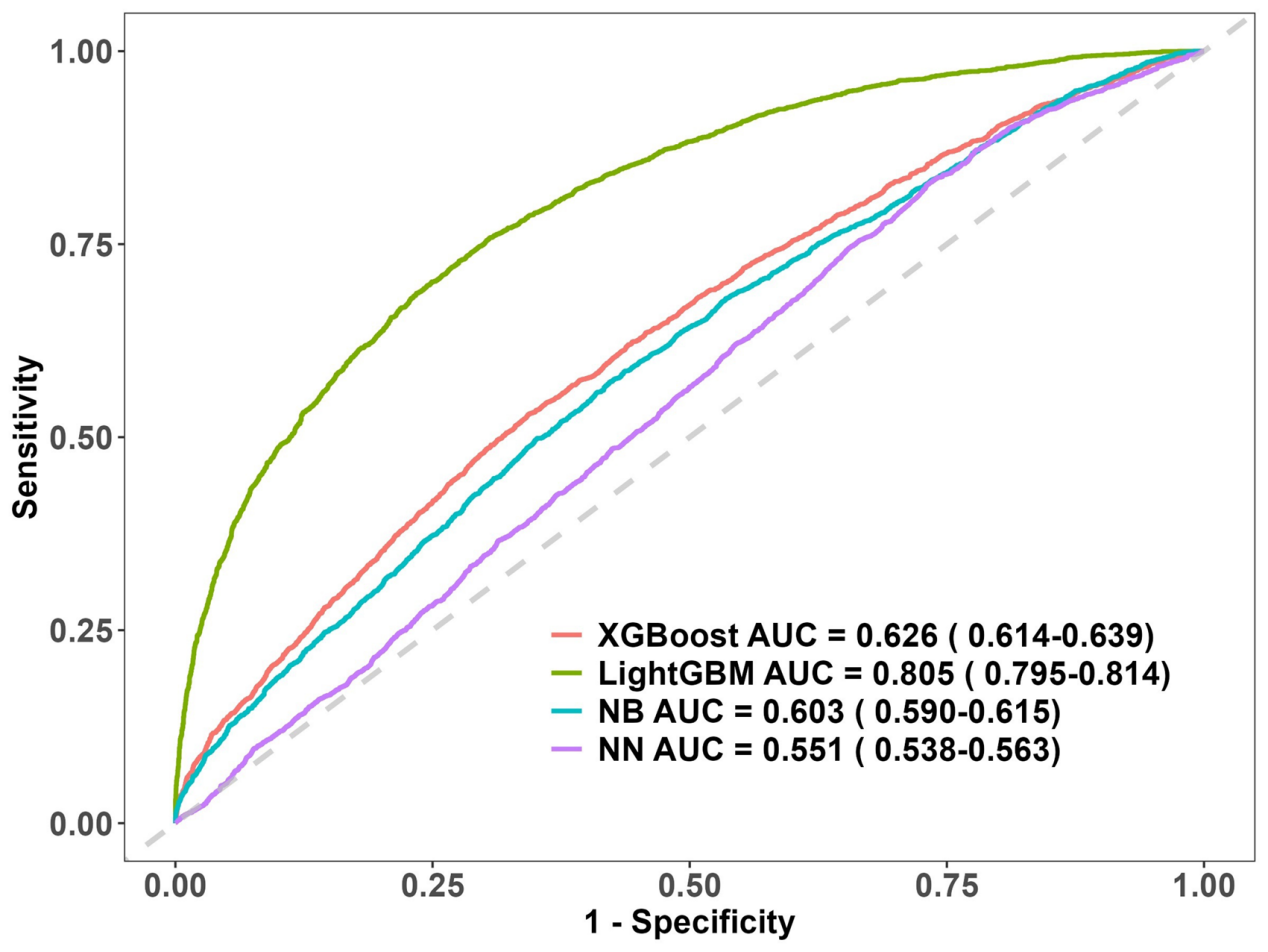
Predictive performance evaluation of ML-Based Predictive Models using ROC curves.

**Figure 5:**
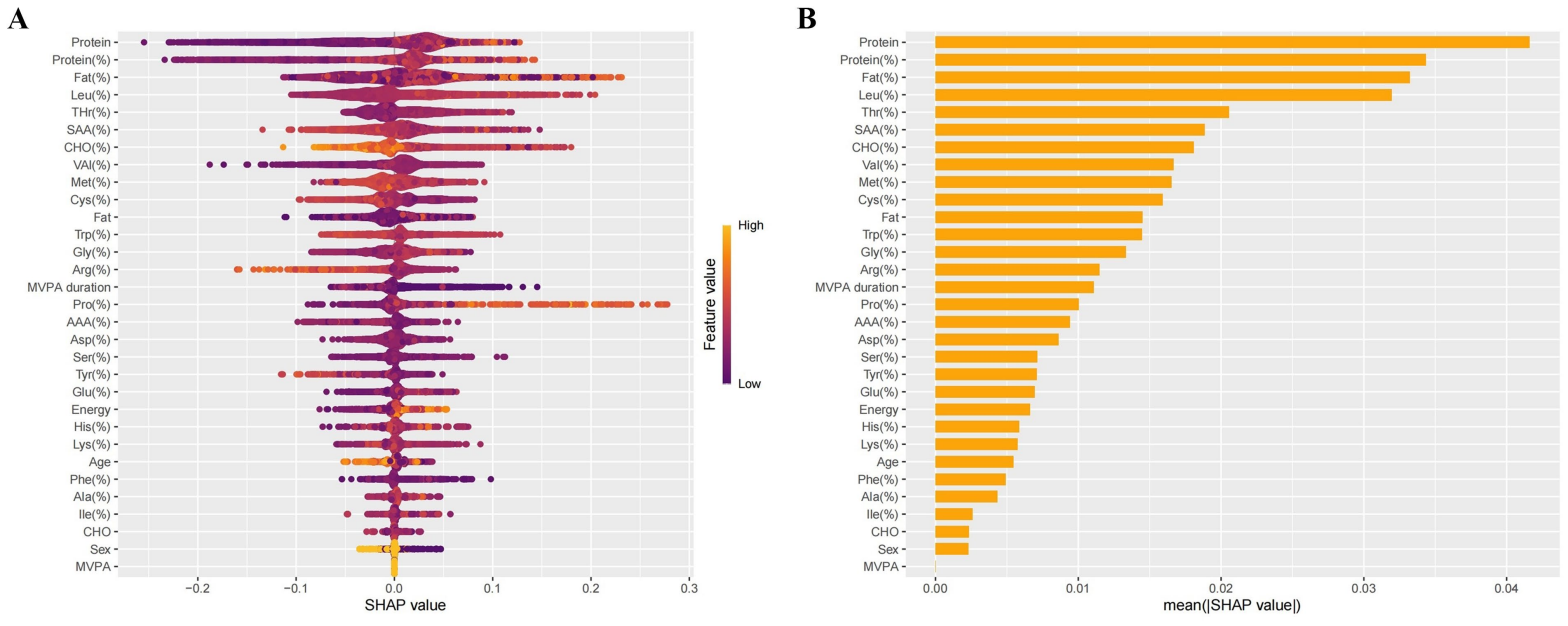
LightGBM model evaluation and interpretation via SHAP analysis. **(A)** Visualizing the direction and magnitude of each feature’s effect (purple: minimum feature range, yellow: maximum feature range). **(B)** Ranking of features by mean absolute SHAP value. The mean absolute SHAP values for the top 10 features are: Protein: 0.0415, Protein(%): 0.0343, Fat(%): 0.0332, Leu(%): 0.0319, Thr(%): 0.0205, SAA(%): 0.0189, CHO(%): 0.0181, Val(%): 0.0167, Met(%): 0.0166, Cys(%): 0.0159.

We evaluated four machine learning models using ROC curves, and found that LightGBM achieved the best prediction effect, with an AUC of 0.805 (95% CI: 0.795 ~ 0.814) (Figure 4). To further interpret this optimal model, this research used the Shapley additive explanation (SHAP) method, derived from game theory, to quantify feature contributions by calculating Shapley values for each variable and identifying the metabolic drivers of overweight/obesity risk. The SHAP result of the LightGBM model indicated that the top amino acids most strongly associated with overweight/obesity risk were Leu, Thr, SAA, Val, Met, Cys (Figure 5).

**Figure 6:**
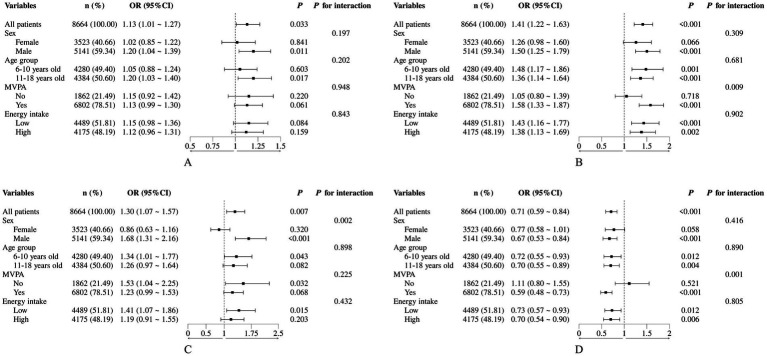
Forest plot showing the stratified association between selected amino acids and overweight/obesity risk: **(A)** Leu, **(B)** Thr, **(C)** Met, **(D)** Cys. Statistical groupings were defined as follows: Sex (male/female), Age group (younger: 6–10 years; older: 11–18 years), MVPA participation (no/yes), and total energy intake (low/high, kcal/day: females, <1,800/≥1,800; males, <2,000/≥2,000).

**Figure 7:**
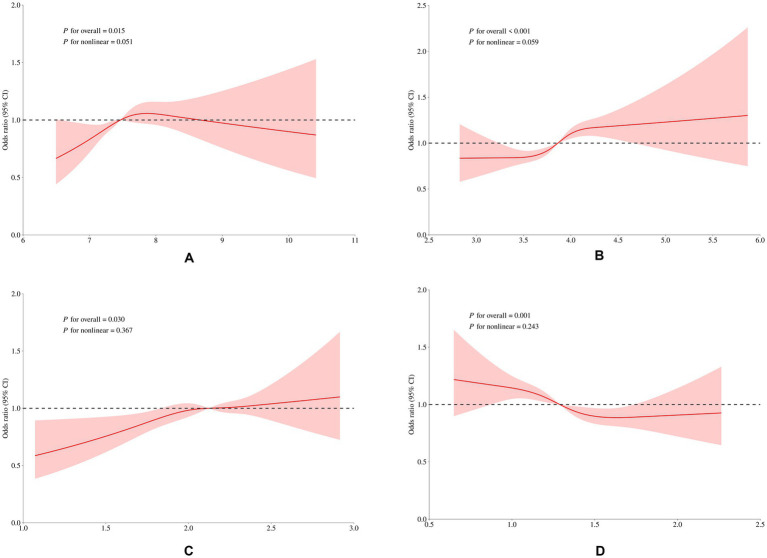
Dose–response relationships between selected amino acids and overweight/obesity risk, analyzed by RCS and adjusted for sex, age, MVPA, MVPA duration, and energy intake: **(A)** Leu, **(B)** Thr, **(C)** Met, **(D)** Cys.

Integrating the outcomes derived from logistic regression and LightGBM, Leu, Thr, Met, and Cys were identified as key amino acids that ranked among the top ten features in the machine learning model and were also significantly related to the risk of overweight/obesity in logistic regression (*p* < 0.05). Sensitivity analyses were further conducted to examine their associations (Figure 6). Stratified analysis showed that the positive associations of these four amino acids with overweight/obesity remained significant in males (*p* < 0.05). Thr and Cys were consistently associated with overweight/obesity across different age groups and levels of energy intake (*p* < 0.05). Leu exhibited a more stable association in the 11–18 years old group, whereas Met showed greater stability in the 6–10 years old group (*p* < 0.05). Interaction analyses indicated that potential confounders, including age, sex, MVPA, energy intake, did not show significant interactions with these associations (*p* > 0.05). According to the result of RCS analysis, there existed linear relationships between the intake of Leu, Thr, Met, and Cys and the risk of overweight/obesity (Figure 7; all *p* for nonlinearity > 0.05).

## 4 Discussion

By integrating machine learning with traditional logistic regression, this study provides the first systematic assessment of dietary amino acid profiles and overweight/obesity risk in a nationally representative sample of Chinese children and adolescents. Our findings identified Leu, Thr, Met, and Cys as key features influencing overweight/obesity risk. Specifically, Thr, Leu, and Met were positively related to higher risk, whereas Cys exerted a protective effect. These findings indicate that specific patterns of amino acid intake may exert critical effects in the progression of overweight/obesity among Chinese children and adolescents, and could indirectly contribute to the early risk of metabolic diseases.

Compared with previous studies, this work addresses several limitations by conducting a comprehensive analysis of amino acid profiles, integrating machine learning with traditional statistical approaches, applying rigorous control of confounders, and performing population-specific analyses. In particular, by leveraging a nationally representative sample that encompasses diverse regional and socioeconomic characteristics, and by incorporating regional dietary cultural variations, our study provides more accurate evidence on the association between overweight/obesity and amino acid intake among Chinese school-aged individuals. These strengths substantially support the generalizability and clinical relevance of our findings.

We found that there exists a positive association between Leu intake and overweight/obesity risk. This finding is consistent with previous evidence. For example, a Chinese study (41) reported that a dietary pattern with feature of high Leu intake, assessed employing a food frequency questionnaire, was closely related to weight gain and elevated BMI. Similarly, an Iranian cross-sectional survey (42) showed that dietary Leu intake was more strongly linked to obesity risk in men than in women, which is in line with our observation that the relation between Leu and overweight/obesity was stronger in males than in females. In addition, a study of children born to mothers with gestational diabetes also found that there exists a positive association between total Leu intake and obesity risk (43). Biomarker studies have further confirmed that higher serum Leu concentrations are significantly correlated with increased BMI (44). Animal experiments also support these findings, showing that a high-leucine diet can reduce insulin sensitivity, increase body weight in rats after a period of high-fat intake (45). The mechanisms underlying the effect of Leu in body weight regulation may involve multiple pathways. First, Leu can activate the mTOR-SREBP1 signaling axis and modulate the level of fibroblast growth factor 21 (FGF21), thereby triggering insulin resistance and promoting lipogenesis within hepatic and adipose tissues (46). Second, branched-chain keto acids (BCKAs), a class of leucine-derived metabolites, may inhibit the tricarboxylic acid (TCA) cycle, leading to mitochondrial dysfunction, reduced fatty acid *β*-oxidation, and disruption of lipid homeostasis. Finally, increased plasma Leu levels may also be linked to higher oxidative stress, which in turn alters the metabolic microenvironment and contributes to body weight dysregulation (47). Collectively, these findings highlight the multifaceted pathways through which Leu influences weight regulation.

Compared with the well-established association between Leu and overweight/obesity, Thr appears to exert dual regulatory effects on energy metabolism. Experimental studies found that restricting Thr intake can significantly reduce fat mass and body weight in mice (48). Consistent with our findings, a large-scale cohort study in China also found that Thr intake was positively related to BMI, body fat ratio, waist circumference, suggesting that higher Thr consumption may elevate susceptibility to the risk of obesity (49). In contrast, other animal studies reported that Thr supplementation reduced body weight, inhibited fat accumulation, and improved lipid metabolism (49–52). Such contradictory results may be explained by differences in experimental conditions, limitations in study design, interactions among nutrients, and potential confounding factors, which may play varying roles across studies and thereby shape the observed association between Thr and overweight/obesity.

Met is an essential SAA, and its intake has been shown to be positively associated with overweight/obesity, which aligns with prior research findings. Experimental evidence indicates that Met restriction increases feed efficiency and energy expenditure, reduces adipose tissue mass, and alleviates obesity in mice (53–56). Similarly, in high-fat diet-fed mice, restricting Met intake significantly attenuates body weight gain (57). The potential mechanisms by which Met contributes to obesity are complex. First, Met restriction may regulate energy metabolism by upregulating FGF21 expression (58). In addition, Met restriction has been shown to suppress the expression of pace-setting enzymes for lipid synthesis, such as fatty acid synthase (FASN), stearoyl-CoA desaturase-1 (SCD1), and acetyl-CoA carboxylase-1 (ACC-1), thereby reducing lipid synthesis. Meanwhile, Met restriction increases the level of adipose triglyceride lipase (ATGL), uncoupling protein 1 (UCP1), and other genes involved in mitochondrial *β*-oxidation, the TCA cycle, and the mitochondrial respiratory pathway, facilitating lipid catabolism and utilization (53). These synergistic mechanisms together form the molecular basis of methionine’s role in obesity development. Cys, another SAA, has shown inconsistent associations with overweight and obesity across studies. Many existing reports suggest that reduced Cys intake can promote adipose tissue browning, inhibit lipogenesis, and increase energy expenditure, thereby reducing body weight (59, 60). However, our study indicates an inverse relationship between Cys intake and overweight/obesity risk. This discrepancy may be explained by several mechanisms. Cys is a key precursor for the synthesis of glutathione, a critical intracellular antioxidant (61). Inadequate dietary Cys intake may compromise antioxidant defense capacity and exacerbate metabolic dysregulation. Concurrently, Cys serves as a precursor for the gas signaling molecule hydrogen sulfide (H_2_S) (62). Sufficient Cys intake ensures normal H_2_S production. This, in turn, improves mitochondrial function, enhances energy metabolism efficiency, reduces fat accumulation, and exerts a protective effect against overweight/obesity. In children and adolescents, a population with unique hormonal and metabolic characteristics, Cys may be more likely to play roles in optimizing metabolic substrate supply and enhancing antioxidant defense. This may result in a protective association with overweight/obesity. Furthermore, this apparent contradiction may be closely related to the metabolic interplay between Cys and Met. Cys can be endogenously synthesized from Met via the transsulfuration pathway. When Met intake is excessive, endogenous Cys synthesis may become relatively abundant or even excessive, even if exogenous Cys intake is low. In such cases, dietary Cys intake may not fully reflect the actual metabolic status of Cys in the body. Consequently, the actual Cys pool may be positively associated with or unrelated to overweight/obesity risk, whereas dietary Cys intake shows an inverse association. This antagonistic interaction between Met and Cys may also partly influence the overall association between SAA and overweight/obesity observed in this study. These findings underscore the necessity of comprehensively considering the metabolic interactions between Met and Cys, as well as their specific roles in different physiological pathways, when investigating the relationship between SAA and overweight/obesity.

Our findings suggest that optimizing dietary amino acid profiles in the diets of children and adolescents—particularly through precise regulation of risk-related amino acids like Leu, Met, and Thr—may serve as a key strategy for preventing overweight, obesity, and related metabolic disorders. Notably, the identification of Thr as a previously overlooked potential risk factor, as well as the metabolic significance of the Met/Cys balance, expands our understanding of amino acid metabolism and offers important implications for both clinical practice and public health policy. However, the implementation of precision nutrition interventions requires careful consideration of individual biological characteristics (e.g., age, sex, genetic background), nutritional and metabolic status (e.g., insulin sensitivity, inflammatory levels), lifestyle factors, and regional dietary culture. Tailoring interventions with these factors in mind will be essential to ensure both safety and effectiveness, while maximizing their impact on the prevention and control of obesity.

Our study is subject to the following limitations. First, the data were derived from a cross-sectional study, causal inferences are inherently limited. Future research employing prospective cohort designs or randomized controlled trials will be needed to more accurately establish the long-term impact of specific amino acid intake on overweight/obesity and to provide stronger causal evidence. Second, although this study adjusted for key variables such as age, sex, energy intake, and physical activity, other potential confounding factors may have been overlooked, such as: (1) metabolic and disease-related factors (e.g., underlying metabolic abnormalities); (2) socioeconomic and family factors (e.g., household income, parental education level, and family history of obesity); and (3) behavioral and lifestyle factors (e.g., psychological status, sleep duration, sedentary behavior, and dietary habits). These unmeasured variables may influence the observed associations by affecting amino acid intake or by directly acting on body weight regulation pathways. As a result, residual confounding may be present. Future studies should incorporate these factors to further validate the conclusions of this research. In addition, although a validated FFQ was used to assess dietary intake, measurement errors and recall bias remain unavoidable. The involvement of both participants and their guardians helped improve data quality. However, in younger children, limited memory capacity and unintentional reporting bias introduced by family assistance may affect data accuracy and reliability. Moreover, day-to-day variation and seasonal fluctuations in diet may introduce estimation errors in nutrient intake. Regional differences in dietary patterns, food varieties, and cooking methods may further contribute to this bias. Despite the use of standardized study designs and statistical methods, information bias related to amino acid intake cannot be completely eliminated. In future research, a multi-method dietary assessment strategy will be employed. This strategy will allow cross-validation and calibration of dietary data. It will also help verify the robustness of the core findings. Furthermore, integrating biomarkers such as plasma amino acid levels with metabolomic techniques will enable cross-validation with dietary data. This approach will provide more direct evidence for elucidating the causal mechanisms linking amino acids to obesity. Finally, this study did not fully explore potential interactions among different amino acids or their synergistic effects with other dietary components, which impairs the comprehensiveness of our conclusions. Future work needs to apply dietary pattern analysis and network analysis methods to investigate amino acid combinations and their interactions with other nutrients in shaping overweight/obesity risk.

## 5 Conclusion

In summary, this study represents the first systematic evaluation of the associations between dietary amino acid profiles and overweight/obesity risk in a nationally representative sample of Chinese children and adolescents. The results consistently identify Leu, Thr, and Met as potential risk factors, and Cys as a potential protective factor. These findings provide a novel and refined perspective for developing dietary strategies to prevent and control childhood and adolescent obesity in China. Accordingly, we recommend emphasizing the overall optimization of amino acid patterns in daily diets. For example, this may be achieved by moderately reducing the intake of red and processed meats, while increasing the proportion of fish, legumes and soy products, and low-fat dairy products as protein sources. Additionally, increasing the consumption of whole grains, nuts, and dark-colored vegetables may help improve overall amino acid balance. In the future, when revising dietary guidelines for children or providing individualized nutritional counseling, incorporating amino acid intake patterns into a comprehensive evaluation of dietary quality may offer a new approach. This approach may support the development of sustainable obesity prevention strategies from a nutritional structural perspective.

## Data Availability

The raw data supporting the conclusions of this article will be made available by the authors, without undue reservation.
